# On Syphilis, Constitutional and Hereditary; and on Syphilitic Eruptions

**Published:** 1853-01

**Authors:** 


### EDITORIAL.

*On Syphilis Constitutional and Hereditary; and on Syphilitic Eruptions.* By ERASMUS WILSON, F.R.S., Author of a Treatise on Diseases of the Skin, etc.—Philadelphia: Blanchard & Lea. 1852. 8vo. pp. 284.

THIS book, which is very appropriately dedicated to Ricord, being in all essential particulars an echo of his teachings, is an illustration of the benefits which a single individual, by a course of rigid experimentation and logical deduction, may confer upon science. Not only are new facts thus added to it, but a school is established whose influence extends far beyond the limits of a *speciality*, and spreads itself over other fields of knowledge. This is what has happened, and is proving true in the case of Ricord. The influence of his system of investigating syphilitic diseases is being felt in every country, and is especially clearing up the few dark points in the pathology of that disease, which remain notwithstanding his labors. Mr. Wilson's work is of the Ricord school, and worthy of its association, being, with the exception of Acton's work, the only one of English origin which suitably represents it.

It is pleasing to see the tribute paid by the author to his master, which is in these terms:—

"The subject of the treatment of syphilis cannot be approached without an eulogium on the genius of Ricord. The published opinions of Ricord are distinguished for their simplicity, their clearness, and their practical application. It is to him that we owe the practical lesson, that the virus of syphilis lies hid in the developing chancre for three days, and that within that limit of time we may destroy the poison in its concealed retreat; that, if it escape us then, mercury must give place to iodine. In this encounter between the wisdom of medicine, and the assaults of a destructive poison, the art is to use and not abuse our remedies; and that power must depend more upon ourselves than upon our teacher. The wrong remedy at a given moment may exasperate instead of checking the disease, and may even serve to perpetuate it in the blood."

This, although far from giving an adequate idea of the importance of his discoveries, or the difficulties which surrounded the subject, is yet gratifying as indicating the progress which his doctrines are making, and pointing to the position which he is destined to assume in the galaxy of bright names which adorn our art, as a star of the first magnitude. By the side of Jenner he may be ranked, without fear of his fame being eclipsed.

Through the beaten field of syphilis we do not propose to follow our author. The unity of the poison—its effects when inoculated—the changes which time makes in its effects—the importance of the abortive treatment—the use of mercury in its earlier, and of iodine in its later stages—all these are too well known to need or bear repetition before well-informed members of the profession.

It is to the more difficult points that our attention is called. Can secondary syphilis be communicated by contamination of the secretions, without giving rise to local symptoms? This is answered affirmatively by Mr. Wilson, and he gives seven cases in proof.

Case seven is essentially this:—"A young man had a venereal sore, which was situated on the inner side of the prepuce. It got well in a few weeks, with the aid of a lotion of the sulphate of zinc, and he was not aware of any secondary symptoms having followed in its train." "Three years afterwards he had gonorrhœa, which lasted two months; and three years later he married." "A fortnight after marriage, the wife, who was a remarkably healthy woman, applied to me in consequence of suffering extreme soreness of the vulva, attended with a discharge. On making an examination, I found the clitoris and labia much swollen, an abrasion, with superficial ulceration of the mucous membrane in several places." This got well under the use of poultices and rest. Secondary symptoms, however, occurred.

This is regarded as an inoculation by the husband, from the sore which he had six years before, and which had left behind no sensible effects.

For ourselves, we cannot adopt this manner of regarding the case. It seems to us far more probable that the disease under which the woman labored was not syphilis, or if so, that it was produced by a fresh infusion. Every surgeon of experience knows how liable parties are to be deceived, and with what caution their statements are to be received as evidence in cases of this kind.

We regard this mode of communication as not proved, but as not by any means impossible. There is one way in which secondary syphilis may be conveyed; it is from the mother to her offspring *in utero*. But, in this case, its effects are well marked, and generally produce abortion. There is no analogy between this and the contaminating the secretions of the genital organs, in the way supposed.

There is another point in which we think Mr. Wilson generalises too hastily and too extensively. It is in regard to the permanence of the syphilitic poison, when once introduced into the system. He believes that it remains during life, and even is felt for several generations, being transmitted hereditarily. That such is the case in many instances cannot be doubted, but that it is invariably or generally so we see no reason to believe. On the contrary, by far the greater number of persons affected for a single time, and well treated, are permanently cured, and no effects of it are subsequently discernable.

The body of the work is devoted principally to the Syphilodermata, or the development of syphilis upon the skin. It is this part which constitutes its chief excellence. It must be admitted that he has succeeded in presenting these in a more natural arrangement, and giving a better view of their relations and modifying causes, than have been offered.

He regards the eruption and sore-throat as analogous to measles, scarlatina, and other exanthemata, as a febrile eruptive movement, resulting from an effort to expel the poison from the system.

If this attack is acute and not too severe, it produces the form of disease known as *Roseola syphilitica*, and which is a simple injection of the skin with dark-colored blood. When these stains subside, they leave behind the copper-colored spots called *Maculæ syphiliticæ*.

When the eruptive force is sufficiently powerful to elevate the pores, the case becomes one of syphilitic *Lichen*, or the papular form of syphilitic eruption. When the force is still greater, several pores and follicular plexuses are elevated together, which constitutes the *syphilitic tubercle*. The distinction between lichen and tubercle is only one of size.

All these spots of roseola, lichen, and tubercle, may be corymbose, circumscribed, disseminated, annular, etc., constituting their varieties. In particular constitutions they become pustular, which adds another complication. The tubercles ulcerating, form syphilitic ulcers of various shapes, sizes, and numbers.

The roseolous form is that which occurs in recent inoculation, the tuberculous in ancient cases.

Such is an outline of Wilson's classification of the Syphilodermata, which he proceeds to fill up in a most concise manner, and which are illustrated by admirable colored plates.

In regard to the hereditary forms of syphilis, we have already said that our author regards them as vastly more frequent and persisting than is generally supposed or suspected. He regards Lupus as of this origin; and intimates that Scrofula, Psoriasis, and Lepra, may be due to the same cause. His remarks on these points are worthy of especial attention.

In treatment, Wilson in the main follows Ricord, using mercury, however, more freely, which, being of London, may be accounted for by his *environment*, although he distinctly admits that the disease may be cured without mercury, and states that this metal is not only useless, but injurious in all cases of tertiary symptoms.

In the work before us, the syphilitic poison is regarded as vastly more subtle, diffusive, and persisting, than has been suspected—as finding its way into the system without local or *primary* symptoms—as presenting itself under forms which have been named distinct diseases, and in subjects removed by generations from its source. The fainter and more delicate tints of the pictures are colored by the hand of a master.

It will be readily understood, that a large part of the human race are, in this view, brought directly or remotely under the influence of the disease, and that it is becoming more disseminated but milder in form. Prevention, therefore, is to be regarded as not less important than cure; and we subjoin some of the more essential points of our author's directions on this head:—

"The first condition of 'taking' the disease is the contact of morbid secretions with some part of the genital organs, either the mucous membrane or the skin: the time requisite for the continuance of the contact is probably very short, particularly in the case of the mucous membane; but *time is necessary*, and upon this circumstance turns the most important of the rules of prevention, namely, *careful washing with soap and water*. This operation should be done well, and immediately after connection, and if it be done well, I think it impossible that absorption can take place, that is, in the male. The female is placed in a position of greater difficulty than the male; the poison, in her case, is conveyed to a situation where washing must necessarily be imperfect. Injection, so far as the vagina is concerned, is her only resource; but so far as affects the external organs, where primary disease most commonly manifests itself, the powers of soap and water will be as potent for her, if properly used, as for the male.

"The best injection for the use of the female, in the case to which I am now referring, is weak vinegar and water. She should first wash with soap and water fully, perfectly, and abundantly, then inject the weak vinegar and water, and then bathe herself outwardly with the same fluid.—[Acids and alkalies possess the power of destroying the poisonous qualities of the syphilitic poison.]—The man should do the same, saving the injection, which is by no means necessary. In both, it is desirable to make water as soon after connection as may be, in order to wash the aperture of the urethra free from any secretion that may have settled there.

"The common situation of development of a venereal sore is among the folds of the prepuce, in the fossa coronæ glandis, and particularly in the smaller and more occult folds of the frænum. It may call for considerable care to expunge any morbid secretions from these situations, and the operation is not one that should be performed negligently or hastily. If a man have a venereal sore on the body of the penis, why, he richly deserves it; for nothing but gross neglect could have allowed the contact of the poisonous secretion for the length of time necessary for absorption, and particularly by the skin, which is not so apt at absorption as the mucous membrane. I have lately seen two instances, in which the sore was developed amongst the hair at the root of the penis. Here it is obvious that the washing had been imperfect, as not having reached that part, and probably the sufferers were not prepared for the development of a sore at so distant a point. This is only a reason for urging the application of the washing the most extensively possible.

"After the washing has been thoroughly effected, the organ should be made perfectly dry, otherwise the moisture left by the ablution may only serve to effect the solution of some undisturbed atom of the poison, and facilitate its absorption.—[In reference to the reproductive powers of the poison, Mr. Acton observes: 'To show the infinitesimal quantity of virus necessary for producing specific effects, one drop has been diluted with a pint of water, and the inoculated fluid has produced a pustule.']

"The power of oil of forming a kind of varnish to the skin, and preventing the contact of moisture for a time, might also be advantageously put in operation as a defence against the syphilitic poison, and as an antecedent, where careful washing is to follow as a subsequent. But it must not be relied upon as a sole defence; nothing should be permitted to interfere with the after-action of the soap and water.

"Where a man is determined to rush into danger, the prepared cæcum of the sheep may be advised as a protection. It is one of great efficiency, and we are occasionally consulted under circumstances which render it necessary that we should be aware of the existence of such a remedy. The unexperienced may deride the suggestion, but the man of the world will appreciate its value."

